# Promoting Occupational Health through Gamification and E-Coaching: A 5-Month User Engagement Study

**DOI:** 10.3390/ijerph18062823

**Published:** 2021-03-10

**Authors:** Chao Zhang, Pieter van Gorp, Maxine Derksen, Raoul Nuijten, Wijnand A. IJsselsteijn, Alberto Zanutto, Fabio Melillo, Roberto Pratola

**Affiliations:** 1 Department of Industrial Engineering & Innovation Sciences, Eindhoven University of Technology, P.O. Box 513, 5600 Eindhoven, The Netherlands; p.m.e.v.gorp@tue.nl (P.v.G.); m.a.derksen@student.tue.nl (M.D.); r.c.y.nuijten@tue.nl (R.N.); W.A.IJsselsteijn@tue.nl (W.A.I.); 2 Fondazione Bruno Kessler, Via Sommarive, 18, 38123 Povo, Italy; azanutto@fbk.eu; 3 Engineering Ingegneria Informatica S.p.A., Piazzale dell’Agricoltura, 24, 00144 Rome, Italy; Fabio.Melillo@eng.it (F.M.); Roberto.Pratola@eng.it (R.P.)

**Keywords:** health informatics, occupational health, social gamification, E-coaching, user engagement, user study, behavior change

## Abstract

Social gamification systems have shown potential for promoting healthy lifestyles, but applying them to occupational settings faces unique design challenges. While occupational settings offer natural communities for social interaction, fairness issues due to heterogeneous personal goals and privacy concerns increase the difficulty of designing engaging games. We explored a two-level game-design, where the first level related to achieving personal goals and the second level was a privacy-protected social competition to maximize goal compliance among colleagues. The solution was strengthened by employing occupational physicians who personalized users’ goals and coached them remotely. The design was evaluated in a 5-month study with 53 employees from a Dutch university. Results suggested that the application helped half of the participants to improve their lifestyles, and most appreciated the role of the physician in goal-setting. However, long-term user engagement was undermined by the scalability-motivated design choice of one-way communication between employees and their physician. Implications for social gamification design in occupational health are discussed.

## 1. Introduction

Many people in modern Western society spend one-third of their time at their workplace on weekdays [1]. As a result, routine behaviors at work (e.g., how long one sits at work, what one eats for lunch and drinks during coffee breaks) should have equal if not greater impacts on people’s health than their private lifestyles. Unfortunately, modern workplaces may causally contribute to several behavioral risks of chronic diseases (e.g., type-2 diabetes, hypertension) due to the high competitiveness and specialization of the jobs. For example, modern knowledge-intensive jobs are arguably a main contributor to the prevalent problem of a sedentary lifestyle [2,3,4], as office workers spend most working hours sitting in front of computers. On the other hand, labor-intensive workers often operate in environments with more health risks, and even though they move more, monotonous physical activities do not help to improve their health status. Furthermore, job-related stress is common in many occupations and it undermines both mental and physical health [5,6,7,8]. As these health issues become increasingly salient, there is a growing awareness that health promotion at workplace is a must. High-impact initiatives include the health promoting workplace (HPW) task force by the WHO [9] and the Total Worker Health (TWH) program by the National Institute for Occupational Safety and Health (NIOSH) [10]. Other organizations and private companies have also started to take more responsibility in health promotion research and practice [11,12,13,14].

Promoting healthy behaviors is a challenging task. It is widely acknowledged among researchers that simply educating people about the importance of health and what to do is not enough [15,16,17]. Without more personalized support, people often fail to translate their health goals and intentions to actions because of temptations, self-control challenges, and bad habits [18]. Furthermore, one-on-one human coaching is not scalable. With the recent advances in big data, sensor network [19] and internet of things, the fourth industrial revolution (also known as Industrial 4.0) may mitigate the limitations of traditional health promotion programs and offer a lever for a qualitative leap in the field of occupational health. A particular promising solution in this technological revolution is the use of mobile gamification systems to support individuals to self-manage their diseases and/or to adopt healthier lifestyles [20,21,22,23]. State-of-the-art applications are sophisticated enough to incorporate multiple behavior change techniques [24] such as goal-setting, self-monitoring, social interactions, and game mechanics to motivate users to reach their goals. Although mobile gamification systems have been researched extensively and demonstrated to be effective in specific contexts [25,26,27], their long-term benefits are still under debate [28] and applications to occupational health remained scarce (but see [29]).

Occupational settings provide some unique challenges as well as opportunities for designing effective social gamification systems. First of all, a company or an organization provides a natural and meaningful context where gamified social competition can be created. Employees can be motivated to compete with their colleagues, friends, or as a team member against other teams in the organization. However, this opportunity comes with a challenge of ensuring *fairness* of the game [30]. Compared with more interest-based communities (e.g., jogging, smoking cessation), employees in an organization can be very heterogeneous in terms of their health status and behaviors they wish to change. For example, a 50-year-old manager with chronic conditions may be content with taking a few more breaks at work, while a young intern in the twenties may have a goal of using the company’s fitness facilities more often. If not designed carefully, the goal heterogeneity may lead to unequal opportunities and/or rewards, which in turn decrease user motivation and engagement [31,32]. While it is possible to create sub-group competitions, it is more interesting to explore whether a creative design can enable users with different goals to compete in the same game (e.g., through asymmetrical game design, see [33]).

Secondly, while *privacy* is of great importance in any gamification design [34], it is even more so for applications in occupational health settings. Given the sensitive nature of many health-related behaviors, employees may not want their colleagues to know what goals they wish to achieve or what behaviors they wish to change (e.g., to lose a certain amount of weight, or to reduce alcohol intake). Furthermore, employees may be even more concerned if their personal health and behavioral data are disclosed to their managers. Research and news stories have shown that personal profile and traces on digital media, e.g., Facebook’s News Feed, can severely influence one’s career prospect [35]. Therefore, protecting privacy is a strict requirement in designing social health gamification in a corporate setting, but it adds challenges for creating a transparent and engaging game.

Thirdly, large organizations usually have one or a few occupational physicians who can potentially enhance the user experience of social gamification systems through human coaching. In this paper, and in the European context, we define an occupational physician as a person appointed by the employer who collaborates with the employer in assessing the risks of a company and carries out health surveillance of workers. Occupational physicians can augment fully automated systems in several ways, including goal-setting consultancy, online diet and exercise coaching, and relapse prevention, and they are usually trusted by employees in the same organization. Digital systems, in turn, give these physicians an opportunity to engage more closely with their clients with manageable additional efforts. It is nonetheless a design and empirical question to what extent company physicians should be involved considering both costs and effectiveness.

In this paper, we present a user engagement study for a social gamification application called DMCoach+, which was developed to address the design challenges and opportunities in occupational health settings. In order to create an engaging game for employees that is fair and privacy-preserving, an innovative two-level design was used in DMCoach+. At the level of *personal challenge*, individual employees were motivated to achieve their personal health goals in the domains of food intake, physical activity, drinking, smoking, and weight management. At the level of *social challenge*, the heterogeneous goals were transformed into a unified point system so that employees in the same organization could compete by comparing their total number of points with each other. The digital game design was augmented by appointing an occupational physician to set up personal goals for each employee at the adoption of the application, and to continuously coach the users digitally through one-way communication during their everyday usage. A 5-month user study with 56 employees from one large organization was conducted to evaluate the application, focusing on the impacts of the two-level game design on behavior change and subjective evaluation of the involvement of the occupational physician. Results showed that while using DMCoach+, approximately half of the participants were able to reach their goals by changing their lifestyle behaviors in positive ways (especially in terms of diet and physical exercise), and many explicitly appreciated the role of the application in the process. Moreover, participants spoke very positively about the involvement of occupational physician in the initial phase of the program, but also acknowledged that the one-way communication with the physician undermined long-term engagement. These findings have important implications not only for the design of DMCoach+ (and similar applications), but also for the research on gamification in occupational health in general.

The remainder of the paper is structured as follows: Section 2 reviews recent relevant works on gamification applications in occupational health settings and clarifies specifically the Unified Health Gamification (UHG) approach. Section 3 discusses the design of the DMCoach+ application and the objectives of the evaluation research. Section 4 and Section 5 report the method and results of the user engagement study. Section 6 concludes the paper with a summary of the findings, implications for occupational health and gamification design, and limitations and future work.

## 2. Literature Review

### 2.1. Related Work on Gamification in Occupational Settings

Already in 2001, an expert committee from the US Institute of Medicine recommended that “work site interventions and evaluations are needed to promote behavioral change […], and increase healthy environments” [36]. The committee also highlighted that such interventions should include multiple persuasive strategies, such as “individual-level attributes, social supports and social norms, family and neighborhood factors, and environmental and social policies”.

A set of promising persuasive strategies are gamification techniques. In gamification, game mechanics are employed outside game contexts in order to motivate participation, engagement, and loyalty [37,38], by leveraging people’s natural desires for autonomy, competence and relatedness [39]. This unique potential to foster (intrinsic) motivation makes gamification techniques promising for increasing engagement with corporate health programs [40].

Over the past decade, many (digitized and gamified) corporate health programs have been successfully employed at worksites, for example, to improve dietary intake [41,42], advertise smoking cessation [43], promote cycling [17], and promote physical activity in general [44]. Two promising persuasive strategies that these studies reported on were applying social dynamics (i.e., motivating others or being motivated by others), and personalization (i.e., tailoring of the intervention to personal preferences). Millonig et al. [17] found that social dynamics had a very strong effect on participants, suggesting that emotional aspects (e.g., team spirit, and fun) have greater potential to encourage participants to adopt healthier routines than more rational strategies (e.g., warning participants that their current behavior yields negative health outcomes). Additionally, Oenema et al. [41] argued that the positive impact of their personalized, web-based intervention on dietary intake may be partly explained by the perceived personal relevance and individualization of the information. The meta-analysis by DeSmet et al. [45] supports this claim by recognizing that personalized interventions are more effective than their non-tailored counterparts.

Recent studies have tried to isolate aspects of corporate health programs at a more fine-grained level, in order to find the exact mechanism that triggers behavior change in its participants. For example, it has been found that in the context of a corporate health program, computer-tailored coaching messages do strongly influence the behavioral intentions that people make, but further work is needed to let these nudges trigger actual behavior [46]. Furthermore, it has been demonstrated that particularly tangible rewards may be adopted to engage participants in a corporate health program, although further research is needed to reach consensus on the type of reward that is most effective [47]. This study aims to uncover particularly the social aspects that trigger behavior change in corporate health programs by evaluating a gamified intervention that fosters social interaction and includes personalized E-coaching.

### 2.2. Unified Health Gamification

We have outlined that privacy and fairness are two issues that can greatly affect user engagement in point-based gamification systems in occupational settings. To address these issues, Unified Health Gamification (UHG) has been proposed as an extension of gamification to enable the inclusion of points from different activity types on social leaderboards [44]. With a UHG design, it would already be possible to have social leaderboards which integrate points based on eating healthily with those based on performing physical activities. However, in previous works, there has been just only one level of game rules. Those rules defined statically the number of points that a user with a given role would get for performing a certain type of activity. For example, the point system used in [44] is shown in Table 1.

Unlike more simple games, which dictate to all users that one step is worth one point, UHG games respect the autonomy of users by also letting them perform other activities like spinning or swimming sessions. Moreover, UHG has been designed to also reward less fit people (e.g., elderly) with feelings of competence in the social health game as they would have a fair chance of winning too. Previously we tested this approach by implementing social leaderboards which integrated all points produced by such rules. The app implementation showed besides leaderboards also newsfeed items about the points that colleagues were contributing for the team. Initial tests demonstrated positive health outcomes, but further analysis was still needed.

Admittedly, a role-based approach (as illustrated by the rules from Table 1) is not as powerful as an approach where game rules can be tailored at the level of individual users. Such individual tailoring may however be desirable in programs where coaches want to take into account the individual strengths and limitations of users. Furthermore, in the occupational setting, some users may consider it too confidential to disclose to colleagues that they are being coached on weight, glucose levels, etc. In the plain application of UHG however, it is made fully transparent to peer players when another player scored points, and based on which rule. This limitation of UHG is addressed in our current work.

### 2.3. Transtheoretical Model of Behavior Change

The Transtheoretical Model (TTM) provides a good theoretical basis for evaluating users’ progresses in achieving their behavior change goals when using health gamification systems. Furthermore, called the stage model, TTM was originally developed to integrate different theoretical approaches of changing addictive behaviors (e.g., smoking), but its most notable contribution is the identification of different stages in behavior change processes [48]. A behavior change process (e.g., smoking cessation) is assumed to follow the order of pre-contemplation (not recognizing smoking as a problem), contemplation (being awareness of smoking as a problem), planning (planning to quit smoking), action (actively controlling one’s urge to smoke), and maintenance (maintaining the a habit of not smoking), but the model allows relapses to an earlier stage. In the context of gamification research, TTM has also been adopted as part of a framework to understand long-term engagement of users [49].

## 3. Design of DMCoach+ and Evaluation Goals

### 3.1. Overview of the Design

**Figure 1:**
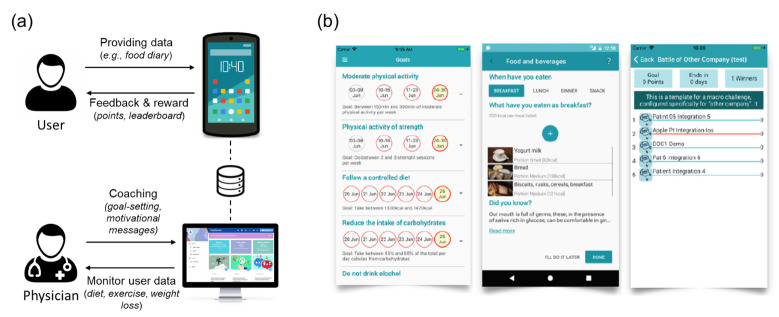
Overview of the design of DMCoach+. (**a**) System architecture; (**b**) Screenshots (left: goals and progress; middle: food diary input; right: leaderboard of the social gamification.

DMCoach+ was developed as a personal health gamification system to be used in occupational settings. The original target users were people with type-2 diabetes who need to change their lifestyles to manage the disease. However, the use case was generalized to healthy adults who pursue a more active and healthy lifestyle in order to prevent type-2 diabetes and other chronic diseases. Figure 1a illustrates the overall architecture of the application. At the user (i.e., coachee) side, users interact with the DMCoach+ mobile app by providing data to the system relating to the goals assigned to them (e.g., reporting food intake, see Figure 1b, middle). They can also monitor their own progress and review the points they earn and their ranks in the social competition (Figure 1b, left and right). At the physician (i.e., coach) side, occupational physicians manage the users through a data dashboard. They also help the users to set their goals and motivate them to achieve the goals. The DMCoach+ application employs a number of theory-based behavior change techniques [50], including *provide information*, *prompt goal-setting*, *prompt self-monitoring*, *providing contingent rewards*, *provide feedback on behavior*, and *provide opportunities for social comparison*. Below we discuss the two most important features in details, the two-level game design and the involvement of occupational physicians.

### 3.2. Two-Level Game Design

In DMCoach+, we aimed for a more personalized UHG approach than what Section 2.2 describes. Furthermore, we aimed for a design that was more conservative from the privacy point of view. Our core design change resided in introducing an additional level in the game rules (i.e., the rules by which points are rewarded to a user):

- *Personal challenge*: The first level of game rules assigns points based on achieved users’ personalized health goals that are defined together with their company physician. Points at this level stimulate users to reach a certain number of target points in each month (e.g., 100).
- *Social challenge*: The second level of game rules assigns bonus points each time a user scores points in the personal challenge (first level). Points at this level are put on a social leaderboard. However, for the sake of privacy, it is not disclosed in the social newsfeed of the app implementation any of the specific reasons why a colleague scores points.

The two-level game design enables a social competition among employees in the same organization for being the most compliant to their personal health goals. Some users may be assigned to goals related exclusively to physical activity and food intake while others can have goals relating to alcohol consumption. The system also allows goals to be heterogeneous in terms of their temporal granularity. For example, besides concrete and short-term goals for performing certain actions (e.g., daily consumption and exercises), users can have long-term weight-loss goals and the application would reward them for incremental weight losses on the shorter term. With these features, DMCoach+ can be easily incorporated into any organization’s health promotion programs, where the virtual points can be translated into more tangible rewards for the employees (e.g., small financial rewards, social recognition, etc.).

### 3.3. Involvement of Occupational Physicians

DMCoach+ was designed in a way that occupational physicians play a pivotal role in its operation. During the initial phase of user enrollment, they create the account for each user and set the personal health goals for them. Their expertise is called upon in order to provide personalized challenges at level-1 of the game, and also to make the level-2 competition fair for all user in the same game (e.g., all employees in the same organization). In addition, the physicians use the dashboard to monitor the data provided by the users and they have the option to send them motivational messages. Users are not able to reply to the messages. This one-way communication was designed based on a previous interview with several occupational physicians, where they expressed their preferences. While this design obviously avoids time-consuming chat interactions and thereby optimizes physician efficiency and enables scalability, we were fully aware of the possibility that it might undermine long-term engagement.

### 3.4. Evaluation Goals

In order to evaluate the novel design elements in DMCoach+, we conducted a 5-month user engagement study. The objectives of the evaluation are three-fold:

1. To observe the temporal change of user engagement in a natural occupational health setting over a relatively long period: *How does user engagement change over time?*
2. To study the behavioral impacts of the application on real users: *Do users make actual progress towards their personal behavior change goals? Do they perceive a positive role of the application in their behavior change processes?*
3. To evaluate the designed level of involvement of the physicians: *How do users evaluate the involvement of the physicians in different phases of the program? Do they perceive the one-way communication design negatively in terms of long-term engagement?*

## 4. Method

### 4.1. Study Design and Context

The user study was an observational field study where participants used the DMCoach+ application for about 5 months in their natural working and home environments. This was followed by an evaluation phase employing a mixed method: we interviewed participants to understand their experience qualitatively, but also examined the behavioral impacts of the application through questionnaires.

The study was set up as a health promotion program at a Dutch university. The university is one of the 14 research universities in the Netherlands and it has around 3200 employees, including doctoral students. Most employees are highly educated, and many are relatively accustomed to digital technologies. As with any universities or research institutes, occupational health can be a salient problem, mainly because of the long hours of sitting in the offices and the high pressure of working in academia. At the university level, there are occasionally short-term health and vitality programs that promote healthier lifestyles among employees, but there is no centralized digital application to support health promotion on a more regular basis. Because the occupational physicians at the university are contractors and they do not usually take part in supervising personal lifestyles, we appointed a professional lifestyle coach from the same region to function as an occupational physician in this specific study.

### 4.2. Participants

Employees at the university were recruited by the researchers through advertisements of the health promotion program. They were promised to become test users of a new mobile-health application and be supported by a professional lifestyle coach. Sixty-one university employees signed up for the study, but 5 withdrew at various stages of the study and 3 did not complete the surveys, leaving 53 participants to be included in the data analyses. For compensation, participants were rewarded a self-tracking wristband (Xiaomi Mi Band 3), which was also used for tracking physical activities during the study.

The eventual sample consisted of 18 females and 35 males and they were between 25 and 57 years old (mean age = 32.7). About half of the participants were PhD candidates (56.6%), and others held positions as non-scientific staff (20.8%), PD Engineering trainees (13.2%), post-doctoral researchers (5.7%), and tenured scientific staff (3.8%). The sample was highly educated, including 6 with a doctoral degree, 41 with a master’s degree, 1 with a bachelor’s degree, 2 with a degree from universities of applied sciences (HBO), and 3 with a secondary vocational education. About half (54.7%) had previously used one or more mobile-health applications before the study. For those who had experience, 55.2% stated that their application(s) helped them to achieve their goals, 31% considered the benefits limited, while 13.8% reported that the applications did not help at all.

### 4.3. Procedure

The study was run between July and December 2019. In July and August, participants who signed up were invited to an introduction meeting about the study in small groups of 2–3 people. The introduction meeting was held in a usability lab and it lasted for about 30 min. At the start, a researcher explained the study to the participants through an 8-min presentation and participants were given opportunities to ask questions. They proceeded to read and sign two consent forms, one about general information of the study and one more specifically about privacy and data management. Next, participants were separated to follow three procedures individually. One participant was invited to a separate room to discuss with the occupational physician their current lifestyle and what goals they wished to achieve in the next 5 months. The physician also created a DMCoach+ user account for the participant and registered the selected goals in the system through the coaching dashboard of DMCoach+. One participant was guided through the functionalities of the DMCoach+ application and instructed on how to use the self-tracking wristband to track physical activities. The third participant was asked to go through an implementation intention procedure [51], in order to help them to better remember to report food intakes in the application. Specifically, they were asked to specify an “if-then” rule, a context where they would report their food intakes daily (e.g., “Before I go to sleep every day, I will register my food intakes in the DMCoach+ app”). They wrote down the rule on a paper document, imagined themselves performing the behavior for one minute, and then signed the document. The participants were rotated to go through all three procedures before leaving the intake. Finally, all participants were asked to complete an intake questionnaire.

During the 5-month study period, participants were encouraged to use the application actively but did not follow any strict protocol about how frequently they should use specific features. In order to observe natural variations in user engagement, the only request was using the app for at least one month to fully experience it, but they were allowed to stop their usage afterwards. Participants provided health and behavioral data (e.g., weight, food intake, smoking, etc.) to the application on a regular basis, and the data were processed to provide them with feedback on their goal achievements (i.e., for their UHG personal challenge), and also to determine the number of points they received for the social game (i.e., for their second level UHG challenge). The leaderboard of the social game was refreshed every month, and to motivate participants further, the results of two rounds of competition were announced to all participants and top-3 winners were awarded small gifts (water bottles and mugs). The physician could also review participants’ data on the coaching dashboard and was instructed to send participants personal messages based on their performance. The system included several pre-determined message templates, but a physician was also free to compose original messages and no strict rules were given about messaging frequency. The physician also sent monthly elaborated educational messages about changing a specific lifestyle behavior (e.g., avoiding sugary drinks).

The evaluation phase of the study took place in November and December. All participants were asked to complete a final online questionnaire around mid-December. In addition, 12 participants with varying levels of engagement during the study were invited to an individual 30-min semi-structured interview. Two additional participants were invited to a 1-hour group interview session, joined by the physician. Interview questions focused on the perceived benefits of the application in their goal achievements and their experience concerning interactions with the physician. Additional questions were asked about more specific usability issues (e.g., design of the food-diary feature, system notifications, etc.), which are beyond the scope of this paper.

### 4.4. Measurements

In both the intake and the final questionnaire, we used a scale adapted from [52] to determine the “stage of change” of the participants in the behavioral domains of physical activity, diet, alcohol intake, and smoking. For example, for physical activity, participants responded “yes” or “no” to the following 4 statements: *“I am currently paying attention to become physically active”*, *“I intend to pay more attention to become more physically active”*, *I currently engage in regular physical activity”*“, and *“I have been engaged in regular physically activity for the past six months”*. Given the combination of answers, a participant’s stage of change regarding physical activity was categorized as one of the five stages as in TTM [48].

In addition, the perceived benefits of involving the occupational physician were evaluated by the participants in the final questionnaire on two 7-point Likert scales created for the study (1 = *Not at all useful*, 7 = *Very useful*), focusing on short-term (e.g., goal-setting) and long-term (e.g., motivational messages) support, respectively. Based on clues from intermediate feedback, we also asked participants to rate to what extent they found that restricting the communication to one-way (physician-patient) messaging had undermined their user experience and long-term engagement. By comparing the differences in the stages between the two questionnaires, behavioral impact of the application was assessed in terms of progresses of behavior change for each of the participants.

Besides the primary measure, the intake questionnaire also measured demographics and participants’ prior experience with similar digital applications. The final questionnaire also included questions about user experience with different features and aspects of the application. These specific usability questions are beyond the scope of the current paper.

### 4.5. Privacy, Data Management, and Ethical Considerations

Participants’ personal data were controlled and processed according to the General Data Protection Regulation under the law of the European Union (GDPR) and Dutch laws for data protection. Personal data were saved on the server of DMCoach+ during the study in order to provide the service to the participants, but were removed after the study, together with their user accounts. Participants were given very detailed explanations about our data management plan, including their rights to modify and remove their data, and they had opportunities to read and accept the privacy policy and terms and conditions of the applications. The contracted professional lifestyle coach only had access to participants’ data during the study and only used the data for the purpose of coaching. In treating the participants in this study, we strictly complied with the ethical principles outlined in the Declaration of Helsinki [53]. The study was also approved by the local ethical committee at the participating university (study number 944).

### 4.6. Data Analysis

We tested whether participants had positive progress in their stages of behavior change in two ways. First, at the group level, for each behavioral domain, a Fisher’s exact test was used to whether the distribution of participants’ stages differed significantly before and after the study period. Second, we examined behavior change progress at the individual level. We computed a change score for each participant in each behavioral domain. For example, the progress score would be 1 if one moved from “action (4)” to “maintenance (5), or -2 from “planning (3)” to “pre-contemplation (1)”. One-sample *t*-test was used to test whether the scores were larger than zero on average, which would indicate positive progress. For the two questions about participants’ communication with the physician, we looked at the distributions of the answers and also used paired *t*-test to examine potential difference between their evaluations for initial engagement and long-term adherence. All statistical tests were two-tailed and an alpha level of 0.05 was used. All analyses were done in *R* programming environment, version 3.63 [54].

Interview data were analyzed following a deductive thematic analysis approach [55]. We first transcribed the data in full and then organized the transcript in Microsoft Excel based on the questions used in the semi-structured interview. The data were then coded and themes were extracted for each interview questions. The identified themes will be reported in the result section and accompanied by quotes from the participants.

## 5. Results

### 5.1. Descriptive Results of App Use

**Figure 2:**
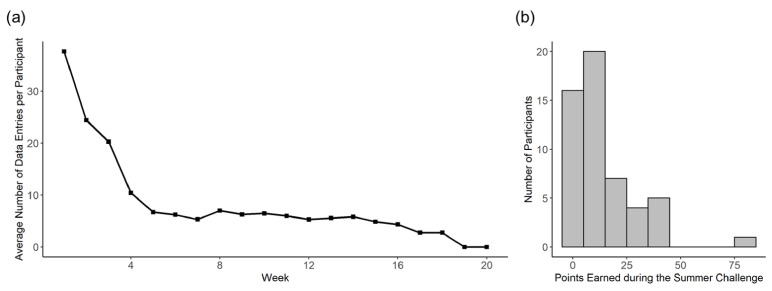
(**a**) User engagement over time as indicated by the average number of data entries per participant; (**b**) Histogram of points earned by participants during the summer challenge

Before examining the main research questions, some descriptive results of app use are shown in Figure 2. User engagement, as measured by the average number of data entries per participant (e.g., a food entry or a physical activity detected by the self-tracking wristband), decreased over the first 5 weeks from 37 to around 5 and then tended to be stable till the end of the study (Figure 2a). During the summer challenge when most participants actively played the social game, the majority earned between 10 and 20 points (i.e., 10 to 20 personal challenges completed in the first two months) (Figure 2b).

### 5.2. Behavioral Impact of DMCoach+

**Figure 3:**
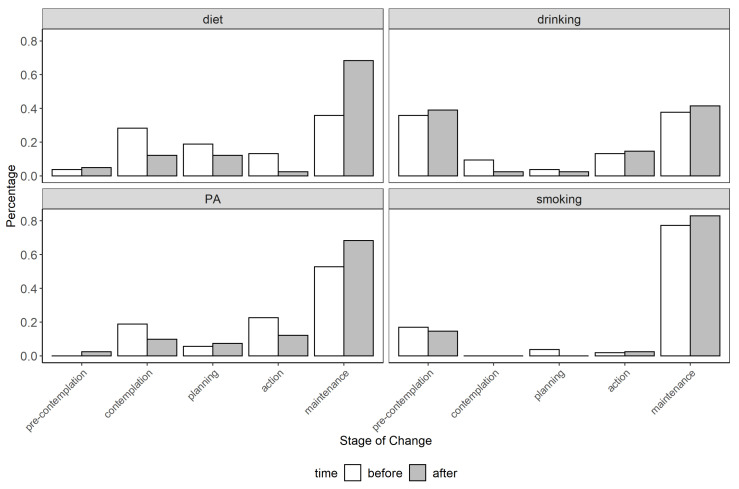
Participants’ stages of change in the four behavioral domains before and after the intervention.

Figure 3 shows the distributions of participants in each of the five stages of change before and after the 5-month health promotion program. Before the study, it was evident that participants had more struggles with keeping a healthy diet than maintaining a good level of physical exercises. Many participants seemed to be aware of their unhealthy diets but were unable to take actions to change them. Relatively few participants had problems with smoking or drinking alcohol excessively (For alcohol drinking behavior, being in the “pre-contemplation” means that participants had no intention to pay more attention to reduce alcohol intake but does not distinguish if they were excessive drinkers. However, based on the feedback from the physician involved in the study, we could know that most participants were not used to drinking excessively), but when they did, they tended to lack motivation to change. Given these baseline patterns, it was most interesting to examine whether participants managed to make progress in doing more physical exercise and following a healthier diet.

For the 41 participants who completed the final questionnaire, visual inspection of the data in Figure 3 suggests that they made some progress in the two behavioral domains that were initially the most problematic—diet and physical activity, indicating positive lifestyle changes while using the application. The positive change before and after the study was significant for diet (*p* = 0.017), but not for physical activity (*p* = 0.272). As expected, since most participants did not set goals for alcohol intake and smoking, no differences in these domains were found.

At an individual level, 15 participants made positive changes in their diets and 13 made positive changes in physical activity. Moreover, 11 and 5 participants made progress in reducing alcohol intake and smoking, respectively. Testing on individual-level progress scores suggested a significant positive progress for diet (*M_diet_* = 0.683, *p* = 0.008), non-significant positive trends for physical activity (*M_PA_* = 0.268, *p* = 0.110) and alcohol intake (*M_drinking_* = 0.463, *p* = 0.134), and no progress for smoking (*M_smoking_* = 0.146, *p* = 0.584).

The quantitative results above were also corroborated by interview data. About half of the interviewed participants stated that they were able to achieve some of the goals that they had set at the start of the study. The achieved goals were from a variety of behavioral domains such as doing more moderate physical exercise, not adding sugar to coffee or tea, reducing consumption of beer, and losing a certain amount of weight. Some participants also explicitly acknowledged the value of the application in supporting them to achieve their goals. The most cited reason was that because specific goals were set in the application, any discrepancies between their behaviors and the goal references resulted in a kind of pressure that motivated them to reduce the discrepancies. This mechanism has been theorized as the self-regulation function of goals in control theory [56] and is reflected in the behavior change technique called self-monitoring [57]. Participants vividly described their own experience of self-regulation, for example:

“I don’t know if I would not have reached my goals without the app, but I did start cycling to work instead of taking the car. I did already sport, but the app is a motivation because you want to register your physical activity. So, it’s easier to go, because it keeps you on track.” (participant 1)

“One of my goals was to not add sugar anymore to my tea or coffee. I think I achieved this goal in two weeks. I just stopped right away. It was not very difficult. I’m still off sugar. I’m eating sweet things, but I don’t add any sugar.[…]In the beginning I was using the app, so I kept trying to stick to this. Furthermore, I think the application in the beginning contributed to this [achieving the goals].” (participant 5)

“I think definitely [the application helped] in the first weeks. Because you want to track your workout and reach your goals. However, if you forgot to track your workout with the band it is very motivating to continue another day. Because it was calculated in the app.” (participant 12)

The application was reported to be helpful also by creating self-awareness for one’s ongoing lifestyle. Many lifestyle behaviors are routine behaviors or habits that people usually pay very little attention to. The fact that the participants had to report these behaviors in the application made them consciously remember and process what they were doing, to reflect on the consequences, and even to gain insights into their existing habits (i.e., self-discovery by self-tracking [58,59]). Consider the following disclosures by the participants:

“I think in general the app was good for awareness, it was good for motivation, but not if there was something beyond your control.” (participant 2)

“The app certainly helped me to lose 6 kilos of weight. This was not something I was able to do before.[…]Yes, I think so [the app was helpful]. Filling in the food helps, and the comments from the coach as well. I’m very sensitive to numbers, so things like the band really helped me as well. The band and the project itself really stimulated me.” (participant 8)

“I wanted to move more, because I was actually not sporting at all. However, I did realize that I was already moving a lot anyway as discovered by the app. I also started smoking and drinking less. I used to drink quite a lot during the week but because I had to report it in the app, I adjusted how much I drank. Furthermore, the most important goal was to lose weight, and I think I achieved all my goals. (participant 9)”

“I wanted to lose weight and I achieved this. The goal of using the app made you think about what you ate and that was a real profit. It made you aware. Yes, I do [think the application helped], because it’s an “ace in the hole” [free translation from Dutch].” (participant 13)

### 5.3. User Evaluation of the Involvement of the Occupational Physician

The perceived benefits of involving the occupational physician were evaluated by the participants through two questions in the final questionnaire. Results showed that participants perceived more benefits of involving the physician in their initial engagement in the health promotion program (*M* = 3.88, *SD* = 1.91) than in the long-term adherence to their goals in the program (*M* = 3.12, *SD* = 1.83), and the difference was statistically significant (*p* = 0.001). The difference might imply that participants were more satisfied with the face-to-face goal-setting meeting at the beginning of the study than the follow-up communications with the physician. We also asked explicitly to participants whether the one-way communication with the physician negatively affected their engagement in the health promotion program, and the majority reported this to be the case (*M* = 5.44, *SD* = 1.61).

Themes identified from interview data also confirmed that participants evaluated the initial goal-setting meeting and the follow-up online communication with the physician very differently. The initial meeting was very well received, and most believed that setting goals together with a health professional was better than doing it by themselves. Some participants believed that the physician was more knowledgeable about health-related behaviors, and thus the goals made could be defined more specifically and at a more appropriate difficulty level (compared to setting them yourself) [60]:

“There was no problem. It was clear. It is better to have a coach otherwise you might set a goal that is too high for you to reach or too low because you want short term gratification. Overall it was nice to have [the coach].” (participant 5)

“Having the coach set a couple of specific and achievable goals was very good. Otherwise I would just be doing something without knowing where I’m going. The goals for calorie intake and workouts were specifically set by the coach. That was good.” (participant 6)

Others cited that setting goals together with another person created stronger commitment to the goals [61] and some social pressure for achieving the goals. This mechanism might have been especially effective because the physician was trusted by the participants and was believed to add a “human touch” to the health promotion program. For example:

“However, I especially think that the fact that there was a real coach involved was a very useful aspect. It gave a feeling of trust.[…]Furthermore, I took the messages more seriously knowing that there was a real coach involved. It felt more trustworthy and someone was putting in the effort so I should do this as well.” (participant 9)

“It was okay. We decided on the targets, of course. The communication with the doctor makes it more serious. It makes you more confident about what you are doing because you are not always sure yourself.” (participant 12)

In contrast, participants were less satisfied with their follow-up online communication with the physician. Among different types of messages sent by the physician, participants especially liked the very detailed health tips sent at the beginning of each month, such as why sugar is bad for health and what to do to reduce sugar intake. However, participants felt that coaching messages were not as personal as they wanted, and even when personal messages were sent, they were not frequent enough. More crucially, many participants explicitly complained that the one-way communication design was not optimal. They argued that the physician could have been more helpful if the application allowed them to initiate conversations with the physician when necessary. For example:

“Yes, and the possibility to send a message back. Because there was a message like “it seems like some of you are not wearing the band anymore”. Furthermore, then I thought, yes, I knew because my dog ate it. However, I could not reply this. So more interaction [would be better].” (participant 3)

“It was one-way so there was not really communication. However, what I received was nice, like I said about the theme messages. Once I received a message that I was doing better than the average person so that was really fun. However, it did not really feel like communication. However, I also don’t really need this. Maybe sometimes to ask a quick question in the app when we got the theme message. So now I googled any questions, but then I was not really sure if it is true or not.” (participant 9)

“It was very frustrating that it was one-way communication. I could not ask questions. It would have been nice to communicate with the coach. The method for this does not matter as long as it’s possible.” (participant 14)

During the group interview, the physician himself also evaluated the initial goal-setting meeting more positively than the follow-up coaching experience. He found that it was difficult to motivate people when only one-way communication was possible. Because participants could not send messages to explain the reasons why they were not providing data, the physician did not have other options but to stop communication after a few reminders were sent.

“Unfortunately, I was not able to receive an answer from you so that was not very nice for me. If a person was not using the app anymore, I stopped sending messages. So I concentrated on those who were still active. However, I found it very hard to motivate people when I was not able to communicate well with them. Two-way interaction would be absolutely beneficial. The first meeting was the best, because I could set goals together with the person. I would have been nice if you would have been able to answer or ask me questions. I think it would improve the overall results.” (the occupational physician)

## 6. Discussion

In this paper, we presented the design and evaluation of DMCoach+, a mobile social gamification system tailored specifically to promote healthy lifestyles in occupational settings. Through an innovative two-level game design, we addressed the challenge of diversity by allowing individual employees to pursue health-related goals that were personally relevant (personal challenge level), and at the same time to participate in a social competition with other employees who might have very different goals (social challenge level). Their privacy was also fully protected by this design as users did not see what goals other users had and were able to achieve. To make sure that the game was fair for all users, an occupational physician was employed to set realistic goals that were similar across users, in terms of difficulty levels. The 5-month user engagement study has demonstrated the general feasibility and applicability of the two-level game design approach: over fifty employees from a Dutch university participated in the same social game while pursuing very different health-related goals.

Moreover, both qualitative and quantitative data suggest positive behavioral impacts of the application among the participated employees. About half of the participants stated during the interviews that they successfully achieved some of their goals selected at the beginning of the study and that the application helped the process by creating awareness and supporting self-monitoring. Measured by the “stage of change” scale, participants also made some positive progresses in the domains of physical activity and eating behavior. Based on statistical tests, the positive progress in eating behavior is very likely to be generalizable to similar contexts.

While these results are encouraging, one should consider them to be preliminary because the attrition between the intake and the final questionnaire might have biased the results [62]. We cannot rule out that the participants who did not respond to the final questionnaire were also the people who were the least successful in changing their behaviors, even though we think this is unlikely. At the very least, when one looks at the data at the individual level, a sizable percentage of the participants moved at least one stage forward in their behavior change journeys. Conversely, it is also possible that some participants stopped using the application after the first two months since they had simply achieved their goals and did not know what else to do. It is thus interesting to explore in future research whether recurrent goal-setting with physicians (e.g., every couple of months) can keep users motivated and engaged for a longer time. In any case, this user study should be followed by more rigorous experiments such as randomized-controlled trials with a formal analysis of drop-out factors.

The one-way communication design was based on suggestions of occupational health physicians, who had requested to minimize their registration burden. However, our empirical results suggest that the communication should be designed differently. Both participants and the physician expressed that the current design undermined the full potential of involving physicians and they would have enjoyed a two-way communication much more. Future iterations of similar systems should enable mutual online communication between users and physicians through the application. Physicians’ workloads can be reduced by automating the most mundane messages such as reminders and simple compliments using smart algorithms.

Another design issue worth mentioning is the trade-off between transparency and privacy. In our two-level game design, we aimed for maximum privacy so that it was not transparent to the users what health-related goals other people had or how other users managed to earn a certain number of points in the social game. Although the user perception of this choice was not systematically examined, we did receive feedback from participants that the clarity of the game rules was compromised because of this design, which might have demotivated some participants. On the other hand, if we maximize transparency, employees may have serious concerns about sharing the information about their health statuses and health-related behaviors within the organization. This ethical issue can be especially profound if more advanced biosensors are used in future health promotion programs that automatically collect and share personal data with the gamification system [19]. In order to develop effective but also ethical gamification systems, a more dedicated study is needed to find the optimal balance between transparency and privacy, perhaps through participatory design [63]. Specifically, we aim to investigate what is more desirable: taking increased transparency as the default, or taking full privacy as the default [64].

There are several limitations in this work. First, because the DMCoach+ application was tested with real users for the first time, some usability issues might have negatively affected user experience and potentially the ability of the study to answer our main research questions. For example, some participants became inactive too early to fully experience the solution. Those participants reported a too high registration burden for food intake and they felt annoyed by automated system notifications. Second, our results may be less applicable to companies or organizations with very different employees or culture. Our participants were highly educated knowledge workers and many were young and were familiar with other e-health applications (e.g., Google Fit, Fitbit). During the interviews, some participants believed that our application should be even more useful for people with less experience with related technologies. This hypothesis can be tested in the future. Finally, our evidence that the DMCoach+ application helped participants to change their behaviors was limited by the use of questionnaire and interview data and the lack of control group. Future research should consider to use randomized controlled trials to test the effectiveness of our approach based on objective behavioral and health measures (e.g., weight loss, hours of physical activity per week, number of cigarettes, units of alcohol, etc.).

In conclusion, our work contributes to the growing literature on the UHG approach [44,46,47] and provides practical implications for all stakeholders involved in workplace health promotion. Our findings show that the two-level game design implemented in DMCoach+ can address the issues of fairness and privacy and motivate employees to change their health-related behaviors. Employers may consider adopting DMCoach+ or similar gamification systems to improve health within their organizations. For occupational physicians, such systems extend their current capacities in support the employees and may fundamentally change the way they work. The message is clear from our study that the active involvement of physicians is crucial for long-term user engagement.

## Figures and Tables

**Table 1:** Illustration of the point system in an UHG game.

Game Rule	Child	Youth	Adult	Elder
Move more than 500 m, but less than 2500 m	40 points	20 points	20 points	60 points
Move more than 2500 m	200 points	100 points	100 points	300 points
Ride a Spinning Bike for 30 min	150 points	75 points	75 points	300 points
…	…	…	…	…

## Data Availability

The anonymous datasets generated and/or analysed during the current study are available from the corresponding author on reasonable request.
